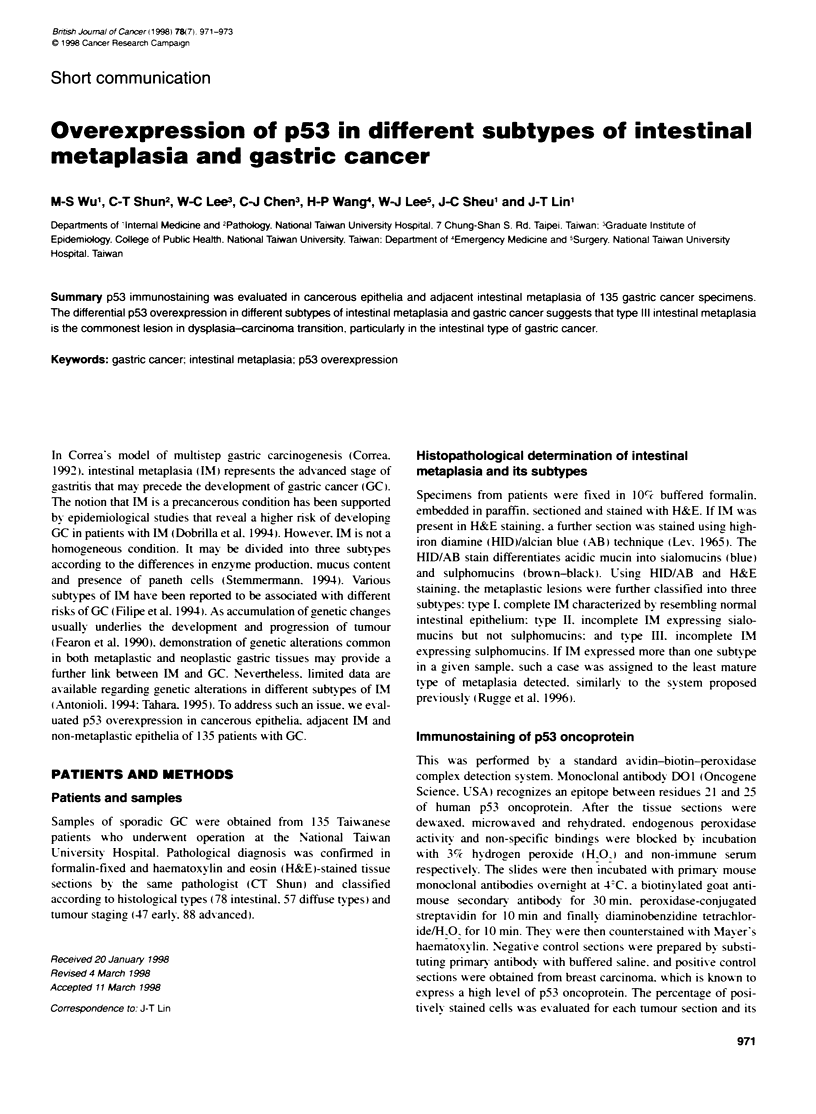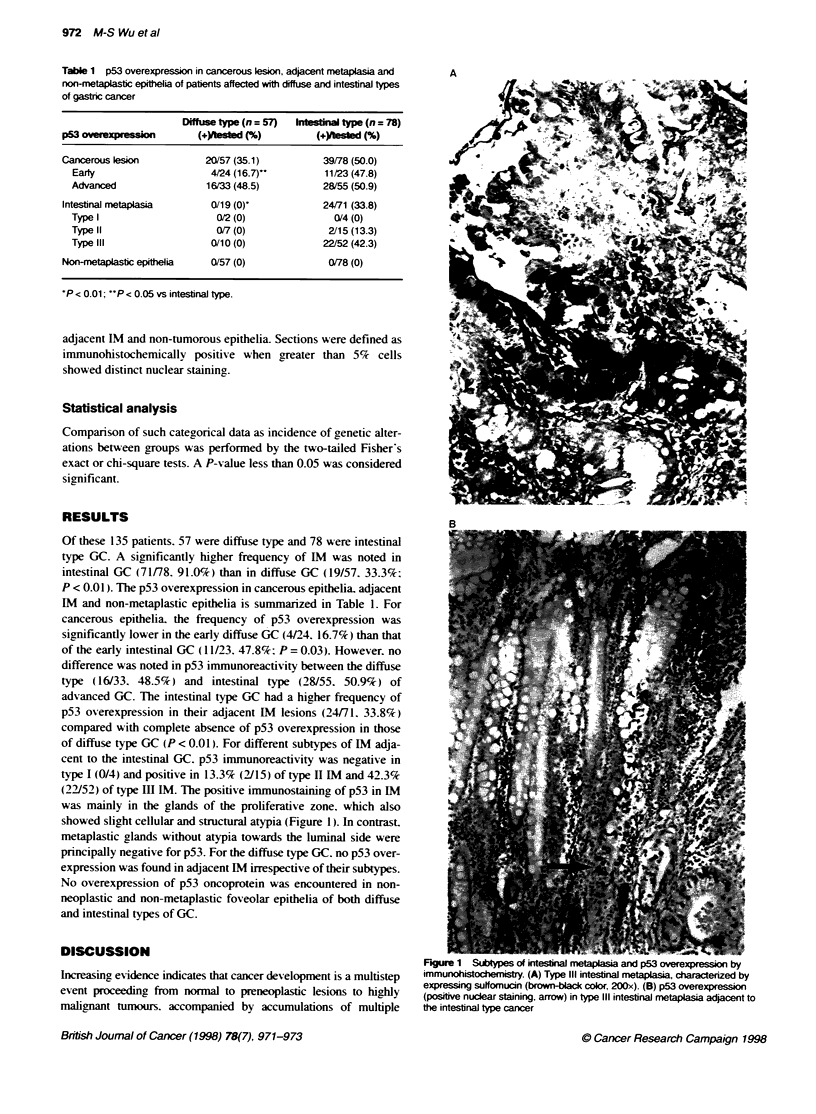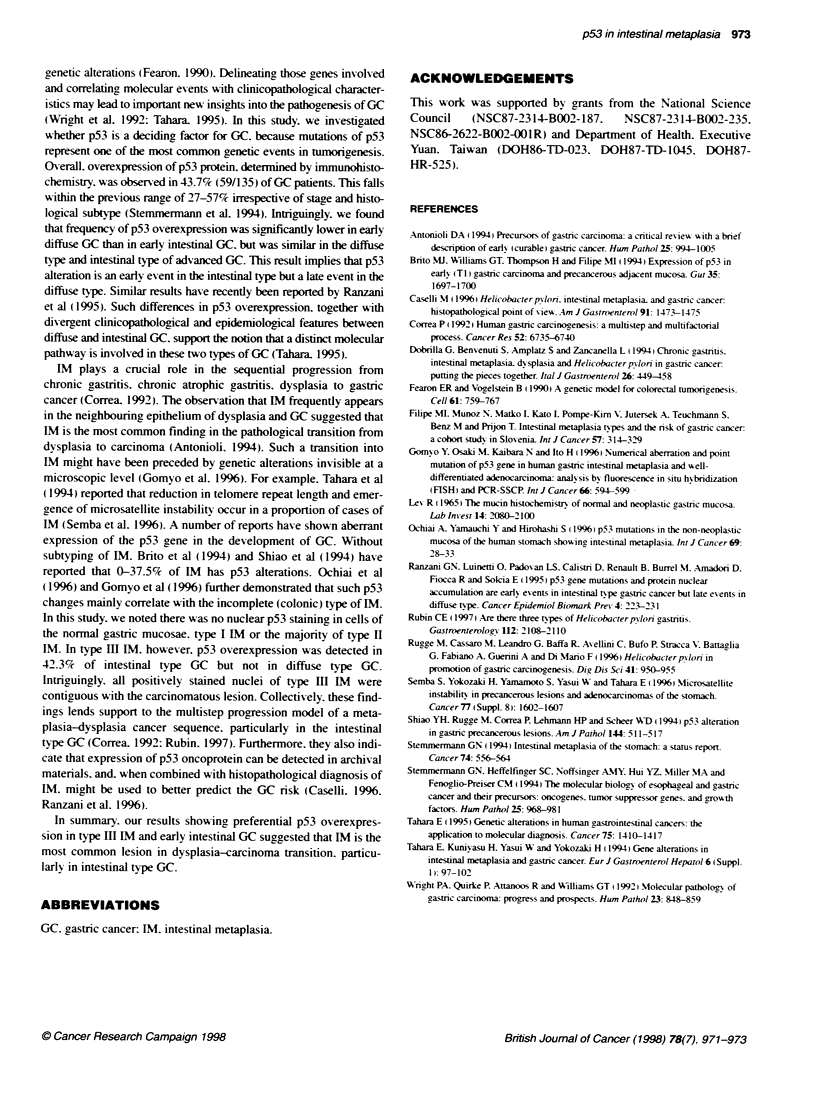# Overexpression of p53 in different subtypes of intestinal metaplasia and gastric cancer.

**DOI:** 10.1038/bjc.1998.611

**Published:** 1998-10

**Authors:** M. S. Wu, C. T. Shun, W. C. Lee, C. J. Chen, H. P. Wang, W. J. Lee, J. C. Sheu, J. T. Lin

**Affiliations:** Department of Internal Medicine, National Taiwan University Hospital, Taipei.

## Abstract

**Images:**


British Journal of Cancer (1998) 78(7), 971–973
© 1998 Cancer Research Campaign

Short communication

### Overexpression of p53 in different subtypes of intestinal metaplasia and gastric cancer

**M-S Wu[1], C-T Shun[2], W-C Lee[3], C-J Chen[3], H-P Wang[4], W-J Lee[5], J-C Sheu[1] and J-T Lin[1]**

Departments of [1]Internal Medicine and [2]Pathology, National Taiwan University Hospital, 7 Chung-Shan S. Rd. Taipei, Taiwan; [3]Graduate Institute of Epidemiology, College of Public Health, National Taiwan University, Taiwan; Department of [4]Emergency Medicine and [5]Surgery, National Taiwan University Hospital, Taiwan

**Summary** p53 immunostaining was evaluated in cancerous epithelia and adjacent intestinal metaplasia of 135 gastric cancer specimens. The differential p53 overexpression in different subtypes of intestinal metaplasia and gastric cancer suggests that type III intestinal metaplasia is the commonest lesion in dysplasia–carcinoma transition, particularly in the intestinal type of gastric cancer.

**Keywords:** gastric cancer; intestinal metaplasia; p53 overexpression

In Correa's model of multistep gastric carcinogenesis (Correa, 1992), intestinal metaplasia (IM) represents the advanced stage of gastritis that may precede the development of gastric cancer (GC). The notion that IM is a precancerous condition has been supported by epidemiological studies that reveal a higher risk of developing GC in patients with IM (Dobrilla et al, 1994). However, IM is not a homogeneous condition. It may be divided into three subtypes according to the differences in enzyme production, mucus content and presence of paneth cells (Stemmermann, 1994). Various subtypes of IM have been reported to be associated with different risks of GC (Filipe et al, 1994). As accumulation of genetic changes usually underlies the development and progression of tumour (Fearon et al, 1990), demonstration of genetic alterations common in both metaplastic and neoplastic gastric tissues may provide a further link between IM and GC. Nevertheless, limited data are available regarding genetic alterations in different subtypes of IM (Antonioli, 1994; Tahara, 1995). To address such an issue, we evaluated p53 overexpression in cancerous epithelia, adjacent IM and non-metaplastic epithelia of 135 patients with GC.

### PATIENTS AND METHODS

### Patients and samples

Samples of sporadic GC were obtained from 135 Taiwanese patients who underwent operation at the National Taiwan University Hospital. Pathological diagnosis was confirmed in formalin-fixed and haematoxylin and eosin (H&E)-stained tissue sections by the same pathologist (CT Shun) and classified according to histological types (78 intestinal, 57 diffuse types) and tumour staging (47 early, 88 advanced).

*Received 20 January 1998*
*Revised 4 March 1998*
*Accepted 11 March 1998*

*Correspondence to:* J-T Lin

### Histopathological determination of intestinal metaplasia and its subtypes

Specimens from patients were fixed in 10% buffered formalin, embedded in paraffin, sectioned and stained with H&E. If IM was present in H&E staining, a further section was stained using high-iron diamine (HID)/alcian blue (AB) technique (Lev, 1965). The HID/AB stain differentiates acidic mucin into sialomucins (blue) and sulphomucins (brown–black). Using HID/AB and H&E staining, the metaplastic lesions were further classified into three subtypes: type I, complete IM characterized by resembling normal intestinal epithelium; type II, incomplete IM expressing sialomucins but not sulphomucins; and type III, incomplete IM expressing sulphomucins. If IM expressed more than one subtype in a given sample, such a case was assigned to the least mature type of metaplasia detected, similarly to the system proposed previously (Rugge et al, 1996).

### Immunostaining of p53 oncoprotein

This was performed by a standard avidin–biotin–peroxidase complex detection system. Monoclonal antibody DO1 (Oncogene Science, USA) recognizes an epitope between residues 21 and 25 of human p53 oncoprotein. After the tissue sections were dewaxed, microwaved and rehydrated, endogenous peroxidase activity and non-specific bindings were blocked by incubation with 3% hydrogen peroxide ($H_2O_2$) and non-immune serum respectively. The slides were then incubated with primary mouse monoclonal antibodies overnight at 4°C, a biotinylated goat anti-mouse secondary antibody for 30 min, peroxidase-conjugated streptavidin for 10 min and finally diaminobenzidine tetrachloride/$H_2O_2$ for 10 min. They were then counterstained with Mayer's haematoxylin. Negative control sections were prepared by substituting primary antibody with buffered saline, and positive control sections were obtained from breast carcinoma, which is known to express a high level of p53 oncoprotein. The percentage of positively stained cells was evaluated for each tumour section and its

**Table 1** p53 overexpression in cancerous lesion, adjacent metaplasia and non-metaplastic epithelia of patients affected with diffuse and intestinal types of gastric cancer

| p53 overexpression | Diffuse type ($n$ = 57) (+)/tested (%) | Intestinal type ($n$ = 78) (+)/tested (%) |
|---|---|---|
| Cancerous lesion | 20/57 (35.1) | 39/78 (50.0) |
| Early | 4/24 (16.7)** | 11/23 (47.8) |
| Advanced | 16/33 (48.5) | 28/55 (50.9) |
| Intestinal metaplasia | 0/19 (0)* | 24/71 (33.8) |
| Type I | 0/2 (0) | 0/4 (0) |
| Type II | 0/7 (0) | 2/15 (13.3) |
| Type III | 0/10 (0) | 22/52 (42.3) |
| Non-metaplastic epithelia | 0/57 (0) | 0/78 (0) |

*$P < 0.01$; **$P < 0.05$ vs intestinal type.

adjacent IM and non-tumorous epithelia. Sections were defined as immunohistochemically positive when greater than 5% cells showed distinct nuclear staining.

### Statistical analysis

Comparison of such categorical data as incidence of genetic alterations between groups was performed by the two-tailed Fisher's exact or chi-square tests. A $P$-value less than 0.05 was considered significant.

### RESULTS

Of these 135 patients, 57 were diffuse type and 78 were intestinal type GC. A significantly higher frequency of IM was noted in intestinal GC (71/78, 91.0%) than in diffuse GC (19/57, 33.3%; $P < 0.01$). The p53 overexpression in cancerous epithelia, adjacent IM and non-metaplastic epithelia is summarized in Table 1. For cancerous epithelia, the frequency of p53 overexpression was significantly lower in the early diffuse GC (4/24, 16.7%) than that of the early intestinal GC (11/23, 47.8%; $P = 0.03$). However, no difference was noted in p53 immunoreactivity between the diffuse type (16/33, 48.5%) and intestinal type (28/55, 50.9%) of advanced GC. The intestinal type GC had a higher frequency of p53 overexpression in their adjacent IM lesions (24/71, 33.8%) compared with complete absence of p53 overexpression in those of diffuse type GC ($P < 0.01$). For different subtypes of IM adjacent to the intestinal GC, p53 immunoreactivity was negative in type I (0/4) and positive in 13.3% (2/15) of type II IM and 42.3% (22/52) of type III IM. The positive immunostaining of p53 in IM was mainly in the glands of the proliferative zone, which also showed slight cellular and structural atypia (Figure 1). In contrast, metaplastic glands without atypia towards the luminal side were principally negative for p53. For the diffuse type GC, no p53 overexpression was found in adjacent IM irrespective of their subtypes. No overexpression of p53 oncoprotein was encountered in non-neoplastic and non-metaplastic foveolar epithelia of both diffuse and intestinal types of GC.

### DISCUSSION

Increasing evidence indicates that cancer development is a multistep event proceeding from normal to preneoplastic lesions to highly malignant tumours, accompanied by accumulations of multiple

A

B

**Figure 1** Subtypes of intestinal metaplasia and p53 overexpression by immunohistochemistry. (**A**) Type III intestinal metaplasia, characterized by expressing sulfomucin (brown-black color, 200×). (**B**) p53 overexpression (positive nuclear staining, arrow) in type III intestinal metaplasia adjacent to the intestinal type cancer

© Cancer Research Campaign 1998

genetic alterations (Fearon, 1990). Delineating those genes involved and correlating molecular events with clinicopathological characteristics may lead to important new insights into the pathogenesis of GC (Wright et al, 1992; Tahara, 1995). In this study, we investigated whether p53 is a deciding factor for GC, because mutations of p53 represent one of the most common genetic events in tumorigenesis. Overall, overexpression of p53 protein, determined by immunohistochemistry, was observed in 43.7% (59/135) of GC patients. This falls within the previous range of 27–57% irrespective of stage and histological subtype (Stemmermann et al, 1994). Intriguingly, we found that frequency of p53 overexpression was significantly lower in early diffuse GC than in early intestinal GC, but was similar in the diffuse type and intestinal type of advanced GC. This result implies that p53 alteration is an early event in the intestinal type but a late event in the diffuse type. Similar results have recently been reported by Ranzani et al (1995). Such differences in p53 overexpression, together with divergent clinicopathological and epidemiological features between diffuse and intestinal GC, support the notion that a distinct molecular pathway is involved in these two types of GC (Tahara, 1995).

IM plays a crucial role in the sequential progression from chronic gastritis, chronic atrophic gastritis, dysplasia to gastric cancer (Correa, 1992). The observation that IM frequently appears in the neighbouring epithelium of dysplasia and GC suggested that IM is the most common finding in the pathological transition from dysplasia to carcinoma (Antonioli, 1994). Such a transition into IM might have been preceded by genetic alterations invisible at a microscopic level (Gomyo et al, 1996). For example, Tahara et al (1994) reported that reduction in telomere repeat length and emergence of microsatellite instability occur in a proportion of cases of IM (Semba et al, 1996). A number of reports have shown aberrant expression of the p53 gene in the development of GC. Without subtyping of IM, Brito et al (1994) and Shiao et al (1994) have reported that 0–37.5% of IM has p53 alterations. Ochiai et al (1996) and Gomyo et al (1996) further demonstrated that such p53 changes mainly correlate with the incomplete (colonic) type of IM. In this study, we noted there was no nuclear p53 staining in cells of the normal gastric mucosae, type I IM or the majority of type II IM. In type III IM, however, p53 overexpression was detected in 42.3% of intestinal type GC but not in diffuse type GC. Intriguingly, all positively stained nuclei of type III IM were contiguous with the carcinomatous lesion. Collectively, these findings lends support to the multistep progression model of a metaplasia–dysplasia cancer sequence, particularly in the intestinal type GC (Correa, 1992; Rubin, 1997). Furthermore, they also indicate that expression of p53 oncoprotein can be detected in archival materials, and, when combined with histopathological diagnosis of IM, might be used to better predict the GC risk (Caselli, 1996, Ranzani et al, 1996).

In summary, our results showing preferential p53 overexpression in type III IM and early intestinal GC suggested that IM is the most common lesion in dysplasia–carcinoma transition, particularly in intestinal type GC.

### ABBREVIATIONS

GC, gastric cancer; IM, intestinal metaplasia.

### ACKNOWLEDGEMENTS

This work was supported by grants from the National Science Council (NSC87-2314-B002-187, NSC87-2314-B002-235, NSC86-2622-B002-001R) and Department of Health, Executive Yuan, Taiwan (DOH86-TD-023, DOH87-TD-1045, DOH87-HR-525).

### REFERENCES

Antonioli DA (1994) Precursors of gastric carcinoma: a critical review with a brief description of early (curable) gastric cancer. *Hum Pathol* **25**: 994–1005

Brito MJ, Williams GT, Thompson H and Filipe MI (1994) Expression of p53 in early (T1) gastric carcinoma and precancerous adjacent mucosa. *Gut* **35**: 1697–1700

Caselli M (1996) *Helicobacter pylori*, intestinal metaplasia, and gastric cancer: histopathological point of view. *Am J Gastroenterol* **91**: 1473–1475

Correa P (1992) Human gastric carcinogenesis: a multistep and multifactorial process. *Cancer Res* **52**: 6735–6740

Dobrilla G, Benvenuti S, Amplatz S and Zancanella L (1994) Chronic gastritis, intestinal metaplasia, dysplasia and *Helicobacter pylori* in gastric cancer: putting the pieces together. *Ital J Gastroenterol* **26**: 449–458

Fearon ER and Vogelstein B (1990) A genetic model for colorectal tumorigenesis. *Cell* **61**: 759–767

Filipe MI, Munoz N, Matko I, Kato I, Pompe-Kirn V, Jutersek A, Teuchmann S, Benz M and Prijon T. Intestinal metaplasia types and the risk of gastric cancer: a cohort study in Slovenia. *Int J Cancer* **57**: 314–329

Gomyo Y, Osaki M, Kaibara N and Ito H (1996) Numerical aberration and point mutation of p53 gene in human gastric intestinal metaplasia and well-differentiated adenocarcinoma: analysis by fluorescence in situ hybridization (FISH) and PCR-SSCP. *Int J Cancer* **66**: 594–599

Lev R (1965) The mucin histochemistry of normal and neoplastic gastric mucosa. *Lab Invest* **14**: 2080–2100

Ochiai A, Yamauchi Y and Hirohashi S (1996) p53 mutations in the non-neoplastic mucosa of the human stomach showing intestinal metaplasia. *Int J Cancer* **69**: 28–33

Ranzani GN, Luinetti O, Padovan LS, Calistri D, Renault B, Burrel M, Amadori D, Fiocca R and Solcia E (1995) p53 gene mutations and protein nuclear accumulation are early events in intestinal type gastric cancer but late events in diffuse type. *Cancer Epidemiol Biomark Prev* **4**: 223–231

Rubin CE (1997) Are there three types of *Helicobacter pylori* gastritis. *Gastroenterology* **112**: 2108–2110

Rugge M, Cassaro M, Leandro G, Baffa R, Avellini C, Bufo P, Stracca V, Battaglia G, Fabiano A, Guerini A and Di Mario F (1996) *Helicobacter pylori* in promotion of gastric carcinogenesis. *Dig Dis Sci* **41**: 950–955

Semba S, Yokozaki H, Yamamoto S, Yasui W and Tahara E (1996) Microsatellite instability in precancerous lesions and adenocarcinomas of the stomach. *Cancer* **77** (Suppl. 8): 1602–1607

Shiao YH, Rugge M, Correa P, Lehmann HP and Scheer WD (1994) p53 alteration in gastric precancerous lesions. *Am J Pathol* **144**: 511–517

Stemmermann GN (1994) Intestinal metaplasia of the stomach: a status report. *Cancer* **74**: 556–564

Stemmermann GN, Heffelfinger SC, Noffsinger AMY, Hui YZ, Miller MA and Fenoglio-Preiser CM (1994) The molecular biology of esophageal and gastric cancer and their precursors: oncogenes, tumor suppressor genes, and growth factors. *Hum Pathol* **25**: 968–981

Tahara E (1995) Genetic alterations in human gastrointestinal cancers: the application to molecular diagnosis. *Cancer* **75**: 1410–1417

Tahara E, Kuniyasu H, Yasui W and Yokozaki H (1994) Gene alterations in intestinal metaplasia and gastric cancer. *Eur J Gastroenterol Hepatol* **6** (Suppl. 1): 97–102

Wright PA, Quirke P, Attanoos R and Williams GT (1992) Molecular pathology of gastric carcinoma: progress and prospects. *Hum Pathol* **23**: 848–859

© Cancer Research Campaign 1998